# Validation of Kidney Donor Profile Index and Estimated Post-Transplant Survival Scores in an Eastern European Transplantation Center—A Seven-Year Retrospective Observational Study

**DOI:** 10.3390/jcm14103540

**Published:** 2025-05-18

**Authors:** Florin Ioan Elec, Tudor Moisoiu, Matei Florin Negrut, Robert Simon, Alina Daciana Elec, Adriana Milena Muntean, Georgeta Horciag, Ana Maria Sitaru, Andreea Liana Rachisan, Gabriel Oniscu, Oana Antal

**Affiliations:** 1 Clinical Institute of Urology and Renal Transplantation, 400006 Cluj-Napoca, Romania; ioan.elec@umfcluj.ro (F.I.E.); tmoisoiu@gmail.com (T.M.); robert.simon@umfcluj.ro (R.S.); dralinaelec@yahoo.com (A.D.E.); munteana2@yahoo.com (A.M.M.); georgetahorciag@yahoo.com (G.H.); antal.oanna@gmail.com (O.A.); 2 Department of Urology, Iuliu Hatieganu University of Medicine and Pharmacy, 400012 Cluj-Napoca, Romania; 3 Biomed Data Analytics SRL, 400696 Cluj-Napoca, Romania; 4 Faculty of Medicine, Iuliu Hatieganu University of Medicine and Pharmacy, 400012 Cluj-Napoca, Romania; 5 Department of Anaesthesia and Intensive Care, Iuliu Hatieganu University of Medicine and Pharmacy, 400012 Cluj-Napoca, Romania; 6 Doctoral School, Faculty of Medicine, University of Oradea, 410073 Oradea, Romania; 7 Department of Pediatrics, Iuliu Hatieganu University of Medicine and Pharmacy, 400012 Cluj-Napoca, Romania; anamariaclaudya@gmail.com; 8 Department of Mother and Child, Discipline of Pediatrics II, Iuliu Hatieganu University of Medicine and Pharmacy, 400012 Cluj-Napoca, Romania; liana.rachisan@umfcluj.ro; 9 Transplantation Surgery Division, Department of Clinical Science Intervention and Technology, Karolinska Institute, 141 86 Huddinge, Sweden; gabriel.oniscu@ki.se

**Keywords:** Kidney Donor Profile Index (KDPI), Estimated Post Transplant Survival (EPTS), predicting scoring system, organ allocation, transplant outcome

## Abstract

**Background/Objectives:** The Kidney Donor Profile Index (KDPI) is an important metric for evaluating the quality of donor kidneys and predicting post-transplant outcomes. The Estimated Post-Transplant Survival (EPTS) score is a tool for estimating kidney transplant candidates’ long-term survival. However, their validity in Eastern European cohorts is yet to be explored. This study aimed to evaluate the predictive accuracy of the KDPI and EPTS in a local cohort. **Methods:** We conducted a seven-year retrospective observational study at a high-volume transplant center in Romania. Data from 353 patients who received kidney transplants from brain-dead donors (DBDs) between 2017 and 2023 were analyzed. The KDPI scores were stratified into <35%, 35–85%, and >85%, while EPTS was stratified into <20%, 20–60%, and >60%. Primary outcomes included one-, three-, and five-year post-transplant graft function as estimated by eGFR, while secondary outcomes involved patient and graft survival rates at one, three, and five years. **Results:** Graft function and survival rates were significantly lower with increasing KDPI and EPTS scores, reinforcing the utility of both scores in clinical decision-making. **Conclusions:** Despite their limitations, KDPI and EPTS remain valuable predictors in our patient population.

## 1. Introduction

Kidney transplantation is the treatment of choice for patients with end-stage renal disease (ESRD). It is associated with better patient survival [1], improved quality of life, and lower costs than conventional renal replacement therapies. Nowadays, the major obstacle facing the field of transplantation is the critical shortage of donor organs; consequently, this leads to an increased use of kidneys previously considered unsuitable for transplantation. The increasing age of donors and the use of organs from Extended Criteria Donors (ECDs) are more and more often recorded [2]. The use of kidneys from suboptimal donors has been associated with a high risk of early graft loss (EGL) and worse long-term graft outcomes [3], highlighting the need for a better assessment of organ quality before transplantation.

Various methods and scoring systems have been developed to assess the quality of donor organs, typically relying on clinical data and, in some cases, histological assessments. Despite the relevance of histological evaluations, their application can be limited due to time constraints, potential sampling errors, and variability between observers, which may lead to unnecessary discards [4]. These factors have contributed to the growing preference for clinical data as a more reliable basis for evaluating organ quality.

Chertow et al. highlighted the significance of donor age as a primary factor for reduced allograft survival [5]. Further investigations confirmed this finding, establishing a clear association between advanced donor age and the reduction in functional nephrons [6,7].

In 2002, the standard criteria donor (SCD) and extended criteria donor (ECD) classification systems were introduced in the United States to enhance the categorization of donor organs. While age played a significant role in this system, the SCD/ECD dichotomy did not lead to a significant improvement and remained a poor predictor of transplant outcomes. This labeling has led to the unnecessary discard of kidneys, primarily due to the negative connotations associated with the ECD classification.

To address these shortcomings, the Kidney Donor Profile Index (KDPI) was developed as a more comprehensive scoring system that provides clinicians with a tool to assess kidney allograft quality on a continuous scale. KDPI integrates multiple clinical variables to determine the risk associated with deceased donor kidneys, thus offering a more accurate and individualized approach to predicting organ function post-transplantation.

Like most European countries, Romania uses a different allocation system based on European guidelines rather than the US-based EPTS/KDPI scores. While both systems consider comparable factors—including waiting time, tissue compatibility, age matching, and immunological factors— the EPTS/KDPI system facilitates optimal matching between high-quality kidney grafts (lowest KDPI) with recipients with the longest expected survival (lowest EPTS). Moreover, the KDPI-EPTS Survival Benefit Estimator, which combines the Kidney Donor Profile Index (KDPI) and Estimated Post-Transplant Survival (EPTS) scores to estimate the potential survival benefit of accepting a specific kidney offer compared to waiting for a potentially better offer, is a clinical tool designed to help transplant professionals and patients make informed decisions about kidney offers. Since in Romania, there is no established risk stratification system for DBDs that can offer comprehensive data to support transplant decision-making, the aim of this study was to investigate the validity of the Kidney Donor Profile Index (KDPI) and Estimated Post-Transplant Survival (EPTS) for predicting short-term and long-term kidney graft outcomes in our transplant population and provide a structured and evidence-based approach to improve transplant outcomes.

Although large-scale studies demonstrate the suboptimal predictive power of KDPI for graft assessment, it remains an accurate predictor of kidney non-utilization [8]. In the USA, two-thirds of the grafts with a KDPI score of 85 or higher are not utilized, raising concerns about the loss of viable grafts [9]. There is no international consensus on using organs with a high KDPI, and due to the organ shortage in Romania, we are increasingly using high-KDPI-score grafts.

## 2. Materials and Methods

**Figure 1:**
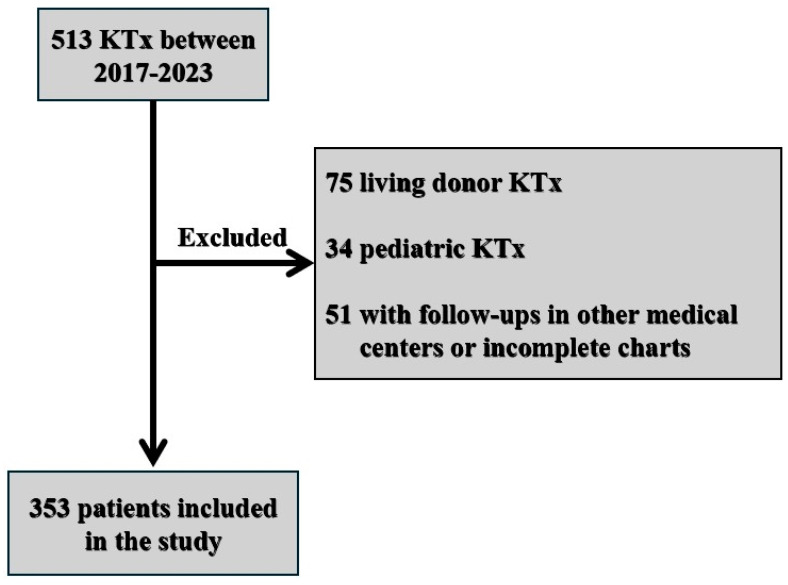
A flow chart of the patients included in the study.

This retrospective, single-center cohort study included 353 adult patients who underwent a DBD kidney transplantation at our institution between the 1st of January 2017 and the 30th of September 2023. Pediatric patients, kidney transplantations from living donors and circulatory-death donors (DCDs), and those lacking complete medical records were excluded from the analysis (Figure 1). In Romania, there is no DCD program. At our center, we do not use grafts from hepatitis C-positive donors and have only transplanted kidneys from Caucasian donors, with no cases involving donors of other races.

This study received approval from the Ethics Committee of the Clinical Institute of Urology and Renal Transplantation (No.3/2024). Given the retrospective and observational design and the anonymization of patient data, the requirement for retrospective written informed consent from participants was waived.

Renal grafts were evaluated based on donor creatinine levels and the Kidney Donor Profile Index (KDPI). The KDPI score was calculated using the online KDPI calculator provided by the Organ Procurement and Transplantation Network (OPTN) [10], stratified into three groups: <35%, 35–85%, and >85%. Patient and graft survival were plotted according to the three KDPI groups.

The Estimated Post-Transplant Survival Score (EPTS) was calculated using an online calculator to assess post-transplant prognosis [11] further. This score includes four clinical parameters: age, time spent on dialysis, previous solid organ transplantation, and diabetic status. Patient and graft survival were also plotted according to the EPTS score groups: <20%, 20–60%, and >60%.

Delayed graft function (DGF), defined as a requirement for dialysis within the first week following transplantation for any indication, was considered a secondary outcome. Another secondary outcome was the renal function assessed using the estimated glomerular filtration rate (eGFR) calculated using CKD-EPI. We studied the causes of patient and graft loss.

Statistical analyses were performed using SPSS Statistics v27.0.0 (IBM Corp., Armonk, New York, NY, USA), Microsoft Excel 365, and GraphPad Prism 8. Continuous variables were expressed as the mean ± standard deviation (SD) when normally distributed and were compared using Student’s *t*-test (assuming equal variances). For non-normally distributed data, variables were reported as median ± interquartile range (IQR) and compared using the Mann–Whitney U test. The normality of data distribution was assessed using the Shapiro–Wilk test, and equality of variances was evaluated with the F-test.

Where appropriate, categorical variables were expressed as frequencies and analyzed using the Chi-square or Fisher’s exact test. Statistical significance was defined as a two-tailed *p*-value < 0.05. Death-censored allograft and patient survival were estimated using the Kaplan–Meier method. Statistical significance for group survival rates was tested using Log-Rank (Mantel–Cox). Multivariable linear regression analysis was conducted to identify the independent factors influencing the glomerular filtration rate (GFR).

## 3. Results

Between January 2017 and September 2023, 513 kidney transplants were performed at the Clinical Institute of Urology and Renal Transplantation Cluj-Napoca. After applying the exclusion criteria, 353 transplants were included in this study. The flow chart of patients included in this study is shown in Figure 1.

The demographics of the donor cohort (*n* = 353) and the distribution according to the three KDPI groups (i.e., <35%, 35–85%, >85%) are shown in Table 1.

Hypertension was identified in 75.6% of the recipients, whereas diabetes was present in 7.6% of the cohort. The median dialysis time before transplantation was two years, with no statistically significant difference between the three KDPI groups. Thirteen patients (3.7%) received a preemptive kidney transplant (KTx), and nine patients (2.5%) had a previous kidney transplant. The one-, three-, and five-year graft function as expressed by the eGFR are shown in Table 2.

We estimated the 5-year patient survival for those on the waiting list (without a transplant) and those transplanted. For all patients, we calculated the survival benefit after kidney transplantation using the KDPI-EPTS Survival Benefit Estimator (11) which ranged between 15.7 and 19.6%. For 132 patients reaching 5-year follow-up, we calculated the actual survival, shown in Table 3. 

**Figure 2:**
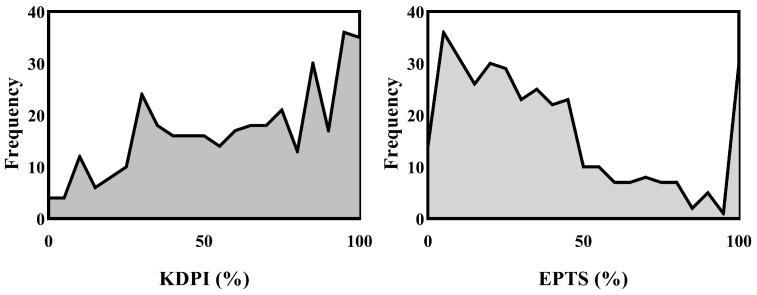
Frequency distributions for KDPI and EPTS scores.

The median KDPI score in our cohort was 65 (IQR: 87–38). The estimated post-transplant survival using the EPTS score showed a median value of 30 (IQR: 52–14). Both the KDPI and EPTS distributions are shown in Figure 2.

**Figure 3:**
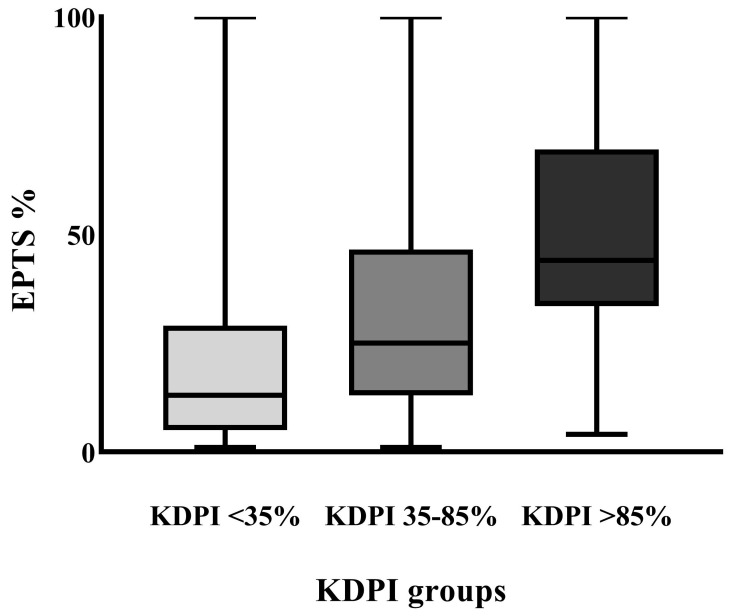
EPTS score in the three KDPI groups.

When we analyzed the distribution of the EPTS in the three KDPI groups, we found statistically significant differences, with a higher EPTS % with increasing KDPI scores (*p* < 0.01, Kruskal–Wallis test), as shown in Figure 3.

**Figure 4:**
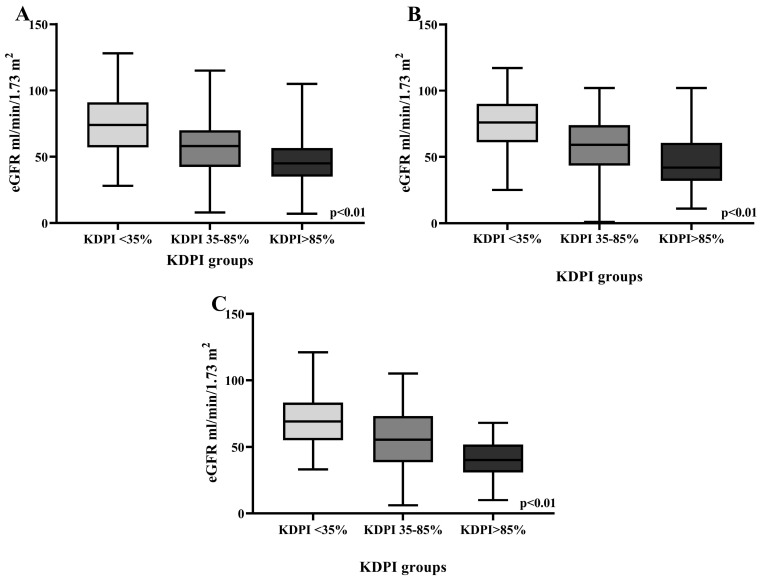
eGFR at one (**A**), three (**B**), and five years (**C**) in the three KDPI groups.

The analysis of eGFR in the three groups at one, three, and five years showed significant differences, with a decrease in eGFR with increasing KDPI scores (*p* < 0.001), as shown in Figure 4.

**Figure 5:**
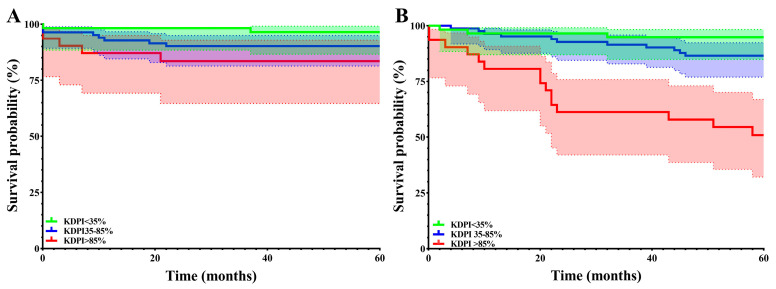
Graft (**A**) and patient (**B**) survival in the three KDPI groups.

We analyzed graft and patient survival at one, three, and five years among the three KDPI groups. We found no significant differences in graft survival but statistically significant differences in patient survival at 3 and 5 years. The results are displayed in Table 4 and Figure 5.

**Figure 6:**
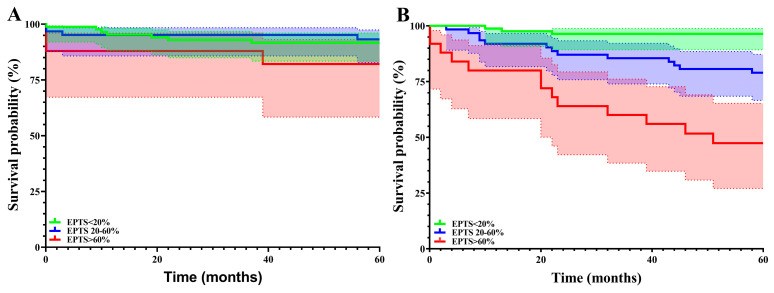
Graft (**A**) and patient (**B**) survival rates according to the EPTS score.

Furthermore, we analyzed the survival rates (graft and patients) according to the EPTS score (%) groups (<20%, 20–60%, >60%) at one, three, and five years. The results are plotted in Table 5 and Figure 6. There was an increase in mortality rate with increasing EPTS scores at all three time points (one, three, and five years post-kidney transplant).

All-cause mortality analysis showed that out of 40 deaths, 18 (45%) were due to COVID-19 infection, of which 8 died during the first wave and 23 in the second wave. Furthermore, thirteen (32.5%) patients were lost to other infections and sepsis, six (15%) to cardiovascular disease, two to complications due to CMV infection, and one due to cancer. When we analyzed all-cause mortality according to EPTS, we found a mortality of 3.53% in EPTS < 20%, 21.7% in EPTS 20–60%, and 50.2% in EPTS > 60%. In the three KDPI groups, mortality rates were 5.2% in KDPI < 35%, 12.4% in KDPI 35–85%, and 49.2% in KDPI > 85%.

## 4. Discussion

The allocation of donated organs is one of the most complex challenges in modern transplant medicine. Clinicians must continuously balance the immediate availability of organs with long-term graft survival, making the Kidney Donor Profile Index (KDPI) a valuable tool in this decision-making process. In this context, KDPI was developed to streamline decision-making and improve the matching of kidneys to recipients with the best long-term prognoses.

Our results showed that KDPI plays a key role in predicting post-transplant survival, showing statistically significant differences in survival across the three KDPI groups (<35%, 35–85%, and >85%) (*p* < 0.01). These findings align with the published literature, highlighting the importance of KDPI in stratifying donor quality, particularly in European populations [2].

In terms of renal function, measured by the estimated glomerular filtration rate (eGFR), our data demonstrated that higher KDPI scores correlated with reduced kidney function at one, three, and five years post-transplant. Recipients with KDPI > 85% showed a significantly lower one-year eGFR than those with KDPI < 35% (approximately 10 mL/min difference). Moreover, the five-year eGFR in KDPI > 85% was significantly lower (40.7 mL/min/1.73 m^2^) compared to the KDPI < 35% group (69.3 mL/min/1.73 m^2^), with a 28 mL/min/1.73 m^2^ difference. This mirrored the findings of Summers et al. [3] and Aubert et al. [9], where higher KDPI kidneys, typically from older and less healthy donors, were associated with reduced long-term renal function.

Despite the differences in renal function, graft survival rates remained relatively good across all KDPI groups. We found no statistically significant difference in graft survival between the three KDPI groups at one, three, and five years. This suggests that while renal function may decline faster with higher KDPI scores, the overall survival of the graft remains acceptable in many cases, suggesting that these organs can still provide substantial benefit, particularly in patients with fewer years of expected survival. Similar findings have been observed by Fabbian et al. (2016), who emphasized that while high-KDPI kidneys are associated with lower function, they can still offer life-saving benefits for patients who may otherwise face prolonged dialysis [12]. Studying graft survival rates beyond 5 years may add further information about the survival perspectives of these renal grafts.

While graft survival showed no statistically significant differences at the three studied time points, patient survival rates among the three KDPI groups differed significantly at three and five years. When we analyzed the estimated and actual median survival rates at five years, we found no statistically significant differences in the low- and median-KDPI groups. In the high-KDPI group, actual mortality rates were higher than estimated. When interpreting these results, we should consider the COVID-19 pandemic, which, as shown by our results, was responsible for almost half (45%) of the deaths in our cohort. Adding that higher KDPI grafts were given to higher EPTS score patients (as shown in Figure 3), the KDPI > 85% group may have increased the vulnerability of these patients, who tended to be older and have higher comorbidities. Furthermore, the discrepancy between estimated post-KTx survival and actual survival in this group may be partly explained by the increased mortality associated with COVID-19 infections, particularly in comorbid and immunocompromised transplant recipients, as shown by several publications [13,14,15]. Overall survival in our cohort was also affected by the high mortality rate in the high-KDPI group.

As we showed in Figure 3, we allocated higher KDPI grafts to higher EPTS score patients. Our findings are consistent with the international literature showing that higher EPTS scores (indicating a lower expected post-transplant survival for the recipient) are often matched with kidneys with a higher KDPI. Studies by Taber et al. (2017) and Coca et al. (2020) found similar trends, emphasizing the principle of “longevity matching”—allocating higher quality kidneys to recipients with a better expected survival to maximize graft utility [16,17]. Recipients with higher EPTS scores (older patients or those with comorbidities) often receive kidneys with higher KDPI because their shorter expected survival times can accommodate the lower anticipated lifespan of the organ. This approach seeks to maximize the utility of each transplant by allocating the best organs to recipients who are likely to benefit from them the longest.

The analysis of the Estimated Post-Transplant Survival (EPTS) score, an essential predictive tool in kidney transplantation, showed that whilst graft survival did not vary significantly among the three EPTS groups, patient survival varied at all three studied time points (*p* = 0.004 at one year and a *p* < 0.001 at three and five years). Our results showed a stratification in survival according to EPTS scores, which mirrored findings from studies conducted in various transplantation centers globally. Clayton et al. (2014) demonstrated that recipients with lower EPTS scores had better post-transplant survival outcomes, validating the use of EPTS in guiding organ allocation [18]. Similarly, recipients with higher EPTS scores were shown to have decreased long-term survival [18].

The high mortality rate observed in the high EPTS group should be interpreted in the context of the COVID-19 pandemic. Whether or not COVID-19-related data should be introduced into the EPTS score is still being determined.

One key purpose of KDPI and EPTS is to match kidneys with a prolonged expected survival (as reflected by low KDPI scores) to recipients with better long-term prognoses (low EPTS scores). Research indicates this strategy is effective, demonstrating that “longevity matching” enhances graft and patient survival rates [19].

While the EPTS and KDPI scores are widely adopted in the U.S., variations in these scoring systems exist globally. Studies conducted in European and Canadian cohorts suggest that while the EPTS score has predictive power in these populations, adjustments may be necessary for regional factors such as donor characteristics and healthcare system differences [16]. Our study suggests that a KDPI-EPTS scoring system may be applied to match kidney grafts with recipients in the Romanian population without additional modifications.

The main limitations of this study included its retrospective, single-center design, which limited the generalizability of the findings to other populations and healthcare systems. Although 353 patients were included in the analysis, the sample size might still have been relatively small when stratified into different KDPI groups, potentially affecting the robustness of the results. Given the limited follow-up in some cases, future research should aim for longer term follow-up to assess the extended impact of KDPI on graft function and patient survival. Tracking patients over 10–15 years post-transplant would provide deeper insights into the long-term viability of kidneys, especially those with high KDPI scores. This study also spanned the COVID-19 pandemic, influencing patient outcomes, particularly in the high-risk KDPI and EPTS groups.

## 5. Conclusions

The KDPI and EPTS scoring systems are valuable tools for kidney transplant decisions. Our study suggests that KDPI-EPTS has the potential to be applied to the Romanian population. A Romanian multicentric survey is mandatory for final validation. Despite the functional limitations of high-KDPI kidneys, they remain viable options when appropriately matched to recipients.

## Figures and Tables

**Table 1:** Demographics of the entire patient cohort (*n* = 353) and the three KDPI groups.

	All Patients *n* = 353 100%	KDPI < 35% *n* = 80 22.7%	KDPI 35–85% *n* = 172 48.7%	KDPI > 85% *n* = 101 28.6%	*p*
Age, years, median, (IQR)	46.7 (55–40)	36 (42.8–28.3)	48 (53–42)	57 (61–52)	*p* < 0.001 ***
Sex					
Females, N (%)	125 (35.4)	31 (38.8)	48 (27.9)	46 (45.5)	
Males, N (%)	228 (64.6)	49 (61.2)	124 (72.1)	55 (54.5)	
BMI	26.4 ± 4.4	24.4 ± 4.5	26.5 ± 4.1	27.6 ± 4.3	*p* < 0.001 **
Donor terminal creatinine mg/dL, median (IQR)	1.03 (1.6–0.7)	0.8 (1.25–0.64)	1.04 (1.47–0.78)	1.29 (1.91–0.83)	*p* < 0.001 ***
HLA mismatches, median (IQR)	4 (4–3)	4 (4.25–3)	4 (4–3)	4 (5–3)	*p* = 0.28 *
History of arterial hypertension, n (%)	267 (75.6)	48 (60)	133 (77.3)	86 (85.1)	*p* = 0.001 *
History of type 2 diabetes, n (%)	27 (7.6)	1 (1.25)	11 (6.39)	15 (17.44)	*p* = 0.001 *
Mean dialysis time before KTx, years, median (IQR)	2 (4–0.9)	2 (4.5–0.75)	2 (4–0.7)	2 (4–1)	*p* = 0.427 ***
Previous KTx, yes, N (%)	9 (2.5)	3 (3.7)	4 (2.3)	2 (1.98)	*p* = 0.735 *
Charlson Comorbidity Index, median (IQR)	3 (2–4)	2 (2–3)	3 (2–4)	3 (3–4)	*p* < 0.05

Abbreviations: N, number; IQR, interquartile range; KTx, kidney transplant; * Chi-Square Test of Independence; ** One-Way Anova; *** Independent Samples Kruskal–Wallis Test.

**Table 2:** The evolution of eGFR after renal transplant for the entire cohort and according to the KDPI groups.

	All Patients *n* = 353 100%	KDPI < 35% *n* = 80 22.7%	KDPI 35–85% *n* = 172 48.7%	KDPI > 85% *n* = 101 28.6%	*p*
EPTS score %, median (IQR)	30 (52–14)	13.5 (28.75–5)	25 (46.75–13)	44 (69.5–33.5)	*p* < 0.001 *
KDPI score %, median (IQR)	65 (87–38)	25.5 (31–11.7)	63 (74–51)	96 (99–92)	*p* < 0.001 *
One-month eGFR mL/min/1.73 m^2^, median (IQR)	44.83 (67.25–28.75)	61.2 (78.6–43.7)	47 (87.7–29.2)	35.5 (45.7–22.6)	*p* < 0.001 **
One-year eGFR mL/min/1.73 m^2^, mean (SD)	58.91 (23.59)	74.6 (22.56)	58 (21.7)	47.5 (20.3)	*p* < 0.001 **
Three-year eGFR mL/min/1.73 m^2^, mean (SD)	60.67 (24.18)	75.9 (21.6)	57.7 (21.9)	47.6 (22.6)	*p* < 0.001 **
Five-year eGFR mL/min/1.73 m^2^, mean (SD)	59.15 (23.65)	69.3 (20.8)	55 (23.7)	40.7 (15)	*p* < 0.001 **

* Independent Samples Kruskal–Wallis Test; ** One-Way Anova.

**Table 3:** Estimated and actual survival rates in the cohort and the three KDPI groups.

	All Patients	KDPI < 35% (80; 22.7)	KDPI 35–85% (172; 48.7)	KDPI > 85% (101; 28.6)	*p*
5-year estimated survival on waiting list (%), median (IQR)	67 (79.3–57.6)	79.3 (84.9–68)	70.6 (79.7–59.2)	60.2 (65.3–52.2)	*p* < 0.001 **
5-year estimated survival if KTx performed (%), median (IQR)	87.5 (93.7–73.9)	94.8 (96–92.2)	89.7 (93.1–83.7)	78.3 (82.2–69.6)	*p* < 0.001 **
5-year estimated survival benefit if KTx performed (%), median (IQR)	17.5 (22.05–13.5)	15.7 (23.8–11.5)	19.6 (24.2–13.7)	17 (18.9–15.8)	*p* < 0.001 **

** Independent Samples Kruskal–Wallis Test.

**Table 4:** Graft and patient survival at 1, 3, and 5 years in the three KDPI groups.

	Total	KDPI < 35%	KDPI 35–85%	KDPI > 85%	*p*
1-year graft survival proportion, N_functional_/N_total_, (%)	339/350 (96.9)	79/80 (98.8)	169 (95.9)	98/101 (97.0)	*p* = 0.48
1-year graft survival time, mean (CI)	11.7 (11.5–11.9)	11.8 (11.6–12.4)	11.6 (11.4–11.9)	11.7 (11.4–12.1)	
1-year patient survival proportion, N_survivors_/N_total_ (%)	332/350 (94.9)	77/80 (96.2)	163/169 (96.4)	92/101 (91.1)	*p* = 0.124
1-year patient survival time, mean (CI)	11.6 (11.5–11.8)	11.7 (11.3–12.1)	11.8 (11.6–11.9)	11.4 (10.9–11.8)	
3-year graft survival proportion, N_functional_/N_total_, (%)	216/229 (94.3)	61/62 (98.4)	103/111 (92.8)	52/56 (92.9)	*p* = 0.278
3-year graft survival time, mean (CI)	34.3 (33.4–35.2)	35.4 (34.3–36.5)	34.0 (32.6–35.4)	33.7 (31.6–35.9)	
3-year patient survival proportion, N_survivors_/_Ntotal_ (%)	200/229 (87.3)	57/62 (91.9)	102/111 (91.9)	41/56 (73.2)	*p* < 0.01
3-year patient survival time, mean (CI)	33.1 (31.9–34.1)	33.9 (32.1–35.9)	34.2 (32.9–35.4)	29.6 (26.6–32.6)	
5-year graft survival proportion, N_functional_/N_total_, (%)	157/172 (91.3)	56/58 (96.5)	75/83 (90.2)	26/31 (83.5)	*p* = 0.09
5-year graft survival time, mean (CI)	55.9 (53.8–58.9)	58.5 (56.4–60.7)	54.9 (51.7–58.3)	53.1 (46.9–59.4)	
5-year patient survival proportion, N_survivors_/N_total_ (%)	143/172 (83.1)	55/58 (94.8)	72/83 (86.5)	16/31 (50.8)	*p* < 0.001
5-year patient survival time, mean (CI)	53.5 (51.8–55.9)	57.6 (54.9–60.4)	55.4 (52.7–58.2)	40.9 (32.8–49.9)	

**Table 5:** Graft and patient survival at 1, 3, and 5 years in the three EPTS groups.

	Total	EPTS < 20%	EPTS 20–60%	EPTS > 60%	*p*
1-year graft survival proportion, N_functional_/N_total_, (%)	339/350 (96.9)	121/124 (97.6)	152/157 (96.8)	66/69 (95.7)	*p* = 0.743
1-year graft survival time, mean (CI)	11.7 (11.5–11.9)	11.9 (11.7–12.1)	11.7 (11.4–11.9)	11.5 (10.9–12.1)	
1-year patient survival proportion, N_survivors_/N_total_ (%)	332/350 (94.9)	123/124 (99.2)	148/157 (94.3)	61/69 (88.4)	*p* = 0.004
1-year patient survival time, mean (CI)	11.6 (11.5–11.8)	11.9 (11.8–12.1)	11.7 (11.4–11.9)	11.0 (10.3–11.7)	
3-year graft survival proportion, N_functional_/N_total_, (%)	216/229 (94.3)	92/98 (93.9)	87/91 (95.6)	37/40 (92.5)	*p* = 0.728
3-year graft survival time, mean (CI)	34.3 (33.4–35.2)	34.5 (33.3–35.7)	34.6 (33.2–35.9)	33.3 (30.4–36.2)	
3-year patient survival proportion, N_survivors_/_Ntotal_ (%)	200/229 (87.3)	95/98 (96.9)	78/91 (85.7)	27/40 (67.5)	*p* < 0.001
3-year patient survival time, mean (CI)	33.1 (31.9–34.1)	35.5 (34.6–36.1)	32.6 (30.8–34.5)	28.2 (24.2–32.1)	
5-year graft survival proportion, N_functional_/N_total_, (%)	157/172 (91.3)	78/85 (91.8)	58/62 (93.5)	21/25 (84)	*p* = 0.245
5-year graft survival time, mean (CI)	55.9 (53.8–58.0)	56.3 (53.6–59.0)	57.1 (53.9–60.2)	51.6 (43.7–59.4)	
5-year patient survival proportion, N_survivors_/N_total_ (%)	143/172 (83.1)	82/85 (96.5)	49/62 (79)	12/25 (48)	*p* < 0.001
5-year patient survival time, mean (CI)	53.5 (51.2–55.9)	58.4 (56.6–60.2)	52.7 (48.6–56.7)	39.7 (30.3–48.5)	

## Data Availability

Data are fully available from the corresponding author upon request.
